# Hybrid System for Local Drug Delivery and Magnetic Hyperthermia Based on SPIONs Loaded with Doxorubicin and Epirubicin

**DOI:** 10.3390/pharmaceutics13040480

**Published:** 2021-04-01

**Authors:** Dorota Nieciecka, Joanna Celej, Michał Żuk, Agnieszka Majkowska-Pilip, Kinga Żelechowska-Matysiak, Antoni Lis, Magdalena Osial

**Affiliations:** 1 Faculty of Chemistry, University of Warsaw, Pasteura 1, 02-093 Warsaw, Poland; joannacelej15@gmail.com (J.C.); mt_zuk@chem.uw.edu.pl (M.Ż.); ai.lis@student.uw.edu.pl (A.L.); 2 Centre of Radiochemistry and Nuclear Chemistry, Institute of Nuclear Chemistry and Technology, Dorodna 16 Str., 03-195 Warsaw, Poland; a.majkowska@ichtj.waw.pl (A.M.-P.); k.zelechowska@ichtj.waw.pl (K.Ż.-M.)

**Keywords:** SPIONs, doxorubicin, epirubicin, magnetic hyperthermia, cytotoxicity, Langmuir-Blodgett technique

## Abstract

Cancer is one of the most common causes of death worldwide, thus new solutions in anticancer therapies are highly sought after. In this work, superparamagnetic iron oxide nanoparticles (SPIONs) conjugated with anticancer drugs are synthesized and investigated as potential magnetic drug nanocarriers for local drug delivery and mild magnetic hyperthermia. We have obtained a hybrid system loaded with holmium and anticancer drugs and thoroughly studied it with respect to the size, morphology, surface modifications and magnetic properties, and interactions with the model of biological membranes, cytotoxicity. We present that nanoparticles having a round shape and size 15 nm are successfully stabilized to avoid their agglomeration and modified with doxorubicin or epirubicin within a controlled way. The number of drugs loaded into the SPIONs was confirmed with thermogravimetry. The hybrid based on SPIONs was investigated in touch with model biological membranes within the Langmuir-Blodgett technique, and results show that modified SPION interacts effectively with them. Results obtained with magnetic hyperthermia and biological studies confirm the promising properties of the hybrid towards future cancer cell treatment.

## 1. Introduction

Over the past several years, there has been a great development of iron oxide-based magnetic nanoparticles application in many scientific fields, including biomedical engineering [1,2], cell labeling [3,4], cancer treatment [5,6,7], magnetic drug delivery [8,9], multimodal imaging [10,11], and Magnetic Resonance Imaging (MRI) contrast agents [12,13,14], due to their unique and diverse biomedical properties. Depending on their composition and surface modification, they recently focused attention on the targeted anticancer treatment and mild magnetic hyperthermia (MH) [15,16,17]. One of the most promising magnetic materials for biomedical use is superparamagnetic iron oxide nanoparticles (SPIONs). Literature shows that modification of the SPIONs, as well as the surface modification with various agents to step closer against the most common type of cancer in women—breast cancer [18,19,20]. The application of SPION allows for simultaneous treatment and monitoring of the tumor growth using and magnetic resonance imaging [21,22,23].

Coating of SPIONs with monoclonal antibodies enables the targeted application of it with reduction of the side effects and controlled release of the drug [24,25,26]. Moreover, incorporation of the lanthanide dopants into the SPIONs puts together magnetic properties of the core with a novel tool in endoradiotherapy [27]. Thus, nonradioactive holmium are replaced with radioactive ^166^Ho radionuclides emitting soft, *β*(*−*) radiation make possible its facile use towards cancer cells [28,29]. For that reason, several studies show successful labeling of the incorporation of radioactive lanthanides into the SPIONs [30,31]. Optimization of the SPIONs properties and linking with anticancer agents enables early treatment of detected cancer and local, targeted treatment, which is a key factor for a favorable prognosis.

Due to the superparamagnetic properties of the magnetic core of SPIONs, it is possible to trigger cancer cells’ magnetic hyperthermia that makes possible by local growth of temperature [32,33]. Within the application of the alternating magnetic field (AMF) with the specific frequency, SPIONs heat up, leading to the destruction of the cancer cells, when injected into the cancer tissue. MHenables heating within the controlled way.

Multiple studies show that multimodal therapy, including the application of SPIONs, gives a synergetic effect in killing cancer cells more efficiently than sole therapy, making spectacular improvement of the therapeutic effects, e.g., greatly extend the lives of patients, even with the most aggressive brain cancers like glioblastoma [34,35], cervical cancer [36] or application in other biomedical fields like to prevent memory dysfunctions or cargo delivery [37,38]. In general, SPIONs have tremendous potential in surface engineering, so they have been widely investigated as a leading strategy towards many diseases [39], especially there, where macromolecule delivery is needed [40] for the facile release of conjugates from their surface [41].

Application of SPIONs as drug carriers delivering conjugated chemotherapeutics seems to be an interesting strategy, especially for decreasing the side effects when the SPION-based conjugate is injected locally. Recently, well-known cytostatics from anthracyclines like doxorubicin (Dox) and its analog epirubicin (Epi), well-known for their effectiveness in cancer therapy, bring attention to application in magnetic nanocarriers [42,43,44,45,46]. Using SPIONs modified with anthracyclines puts together cancer triggering by the drug and the magnetic hyperthermia for a more efficient triggering of cancer cells. Moreover, it reduces the cytotoxicity of Dox and Epi towards the cardiovascular system [47], when the drug is released locally from the drug carrier. Several studies are found in literature focused on the efficient loading of these drugs in the SPIONs. Thi et al. refer to successful SPIONs’ functionalization with Dox, where the drug is entrapped into the polymeric shell [48]. Jalalian et al. describe the successful loading of epirubicin into the SPION-aptamer bioconjugate [49]. Chang et al. while Rao et al. present transdermal application of Epi-loaded SPIONs showing its promising results against cancer [50].

The core-shell functionalization of SPIONs with the drug maintains the desired features of a hybrid like morphology, magnetic properties, and local drug release, especially when the hybrid use is combined with magnetic hyperthermia [51,52].

Due to the many features that make SPION-based anticancer drug conjugates, they are one of the best candidates for local synergetic therapy [53]. In this study, we developed the synthesis of the hybrid based on the holmium-doped SPIONs using the coprecipitation method. The core was coated with citric acid and doxorubicin, or epirubicin via an amide bond as a promising and effective hybrid towards cancer treatment. The obtained hybrid was characterized by various techniques, including Transmission Electron Microscopy (TEM), Dynamic Light Scattering (DLS), Thermogravimetry (TGA), Fourier-Transformed Infrared Spectroscopy (FT-IR), and magnetic hyperthermia (MH). Then, the interactions between the released drug and biological membranes were studied within the Langmuir trough. In addition, cytotoxicity and apoptosis assays were performed to investigate the hybrid anticancer activity towards cells.

## 2. Materials and Methods

### 2.1. Chemicals

Iron (III) chloride hexahydrate FeCl_3_·6H_2_O Aldrich ACS reagent 97%, and iron (II) chloride tetrahydrate FeCl_2_·4H_2_O puriss p.a. ≥99% (RT), were supplied from Sigma-Aldrich (Sigma-Aldrich, St. Louis, MO, USA), holmium (III) chloride hexahydrate HoCl_3_·6H_2_O 99.9% trace metals were obtained from Sigma-Aldrich, 25% ammonia solution NH_4_OH was supplied from POCH (POCH, Wrocław, Poland). Deionized water with a resistivity of 18.2 MΩ cm at 25 °C was obtained using the Milli-Q ultra-pure water filtering system from Merck (Merck, Warszawa, Poland). Citric acid was purchased from Sigma-Aldrich with 94% grade acid. Acetone having analytical grade was supplied from POCH. Epirubicin hydrochloride and doxorubicin hydrochloride European Pharmacopoeia (EP) Reference Standard were purchased from Sigma-Aldrich. KH_2_PO_4_, and K_2_HPO_4_ both with an analytical grade, were purchased from POCH. Human serum albumin was purchased from Thermo-Fisher Scientific Capture Select^TM^ (Thermo-Fisher Scientific Capture Select^TM^, Inchinnan, Scotland).

The following materials were used for cell experiments: McCoy’s Medium, fetal bovine serum, phosphate-buffered saline (PBS), trypsin-EDTA, a penicillin/streptomycin solutions from Biological Industries (Biological Industries, Beth Haemek, Israel), dimethyl sulfoxide (DMSO) (Sigma-Aldrich, St. Louis, MO, USA), CellTiter 96^®^ Aqueous One Solution Reagent (MTS compound) from Promega (Promega, Madison, WI, USA), FITC Annexin V and Propidium Iodine (PI) staining solution from BD Biosciences (BD Biosciences, San Jose, CA, USA).

A human-derived SKOV-3 cancer cell line was obtained from the American Type Culture Collection (ATCC, Rockville, MD, USA) and was cultured in McCoy’s medium supplemented with 10% fetal bovine serum and 1% penicillin/streptomycin. The cells were grown at 37 °C in a humidified atmosphere containing 5% CO_2_.

### 2.2. Synthesis of Holmium-Doped SPIONs

The synthesis of the SPIONs doped with 1% of holmium was performed within the coprecipitation technique as follows.

In three Eppendorfs, solutions containing 426 mg of FeCl_3_·6H_2_O in 4 mL of water, 160 mg of FeCl_2_·4H_2_O in 2 mL of water, and 9.5 mg of HoCl_3_ in 800 µl of water were respectively prepared. Each of them was vortexed until the salt was completely dissolved. The solutions were poured into one beaker. The mixture was heated to 60 °C with continuous stirring at about 1500 rpm, and NH_3_ has been added dropwise until pH 10. Then, the solution turned back-brown, indicating the formation of precipitate containing holmium-hoped SPIONs (called in this work SPIONs). The beaker was then placed on a neodymium magnet to collect the precipitate at the bottom of the beaker. The supernatant solution was poured off, and nanoparticles were washed several times with water to remove unreacted compounds until neutral pH.

### 2.3. Modification of SPIONs with Citric Acid

To overcome aggregation, SPIONs were modified with citric acid that also works as a linker agent with anticancer drugs. To modify the surface of the nanoparticles, 26 mL of 0.05 M citric acid has been added to the precipitate. The pH of the solution was adjusted to 5.2 by dropwise addition of an aqueous ammonia solution. It was heated at 70 °C and stirred with 300 rpm for 90 min to adsorb the citric acid on the surface of nanoparticles. Next, the SPIONs were precipitated with acetone and separated by a magnet. SPIONs coated with citrates (SPION@CA) was rinsed four times with distilled water. This process was designed to remove excess citric acid. Between each wash, the suspension was sonicated using an ultrasonic homogenizer and collected again on the magnet. After several acetone washing, SPIONs were suspended in the Milli-Q water. When the water is added, they are stable, and it is not possible to collect them on the magnet. Based on that procedure, the SPIONs doped with 1% of holmium were obtained. Such a doping level is optimal for maintaining high saturation of magnetization that is crucial for the following use in magnetic hyperthermia [54].

### 2.4. Modification of Nanoparticles with Drug

When the SPIONs were successfully modified with citric acid, the next step was based on the conjugation with anticancer drugs like doxorubicin and epirubicin. At the first step of drug conjugation, 1.5 mM of EDC (*N-*(3-Dimethylaminopropyl)-*N′-*ethylcarbodiimide hydrochloride) aqueous solution (19 mL) and 1.5 mM of NHS (*N-*hydroxysuccinimide) aqueous solution (40 mL) solutions were prepared and added to the beaker with 30 mg of nanoparticles. The mixture has been left on a magnetic stirrer (600 rpm) for about 10 h. In the next stage, 210 mg of the drug (epirubicin and doxorubicin, respectively) dissolved in 102 mL of water was added to the beaker and stirred (also 600 rpm) for another 12 h. The conjugate was decanted onto the magnet, and the solution was poured off the supernatant containing an excess of the drug, as well as an excess of EDC and NHS. Drug-modified nanoparticles were washed with acetone until the red color of the supernatant, indicating the presence of the drug, disappeared. The addition of acetone enabled the collection of the suspension onto the magnet. Thus, it is stable in the aqueous media. After acetone washing, the obtained product was suspended in Milli-Q water to the desired concentration, depending on further studies, e.g., 10 mg/mL in the magnetic hyperthermia analysis, 10 mg/L in the Lamgmuir studies, and 4 µg/mL in the cytotoxicity tests.

### 2.5. Interaction with Biological Membranes

We used 100 mM phosphate buffer (PBS) at pH 5.8 (analog of the tumor cell environment) and at pH 7 (as the environment of normal cells). Nanoparticles, drugs, and conjugates were added to the buffer solution, respectively. The Langmuir trough was cleaned with chloroform and methanol before each measurement. Then, 20–30 µL of chloroform lipid solution like 1,2-dioleoyl-sn-glycero-3-phosphocholine (DOPC), 1,2-dioleoyl-sn-glycero-3-phosphoethanolamine (DOPE), or cardiolipin at a concentration of 2 mg/mL was dropped onto subphase. After 10 min, the solvent was evaporated, and the measurement was started. The experiments were carried out with the compression rate of barrier 5 cm^2^/min, with the final surface pressure 30 mN/m. This value corresponds to the pressure of the membrane occurring in living cells. During the measurements, the curves dependence of the surface area per molecule on the surface pressure so-called Langmuir isotherms were recorded.

### 2.6. In Vitro Cytotoxicity Evaluation

Cytotoxicity studies were performed for nanoparticle dispersions containing: SPION@CA, hybrid linked with doxorubicin (SPION@CA_Dox), hybrid linked with epirubicin (SPION@CA_Epi), and for Dox and Epi as well. Concentrations of nanoparticles (NPs) ranged from 0.5–4 µg/mL (0.05–0.4 µg/mL of Dox and Epi, 10%). SKOV-3 cells were seeded in 96-well plates at a density of 2.5 × 10^3^ cells per well at 37 °C with 5% CO_2_ in a humidified environment. After 24 h, cells were washed with PBS, and various doses of NPs/Epi/Dox compounds were added. Treated cells were incubated for an additional 24 h, 48 h, and 72 h. The MTS assays were performed using CellTiter-96^®^ AQ_ueous_ One Solution Cell Proliferation Assay. The absorbance of the formazan product in wells was measured at 490 nm using a microplate reader (Berthold Technologies, Bad Wildbad, Germany). Results are expressed as the percentage of viable cells in relation to nontreated control cells.

### 2.7. Flow Cytometry Analysis of Cell Apoptosis

Ovarian cancer cells (SKOV-3) (600,000 per well) were treated with SPION@CA, SPION@CA_Dox, SPION@CA_Epi, Dox, and Epi (concentration of NPs—4 µg/mL; Dox, Epi—0.4 µg/mL), 24 h after seeding. After 24, 48, and 72 h of incubation times, detached by Trypsin/EDTA cells were washed twice with cold phosphate buffer (PBS) and furthermore resuspended in 1X Annexin V Binding Buffer. Finally, 5 µL of Annexin V-fluorescin isothiocyanate (FITC) and 5 µL of propidium iodide (PI) were added to the cells. They were incubated for 15 min at RT (25 °C) in the dark. To evaluate the cell death, the analysis of samples was performed using flow cytometry FACSCelesta^TM^ (BD Biosciences, San Jose, CA, USA). The percentage of apoptotic and necrotic cells was determined using FACSDiva software v8.0, BD Biosciences, San Jose, CA, USA.

### 2.8. Statistical Analysis

All presented experiments were carried out at least in triplicate. Statistical analysis was performed using GraphPad Prism Software version 8.0 (GraphPad Software Inc., San Diego, CA, USA). To evaluate the cytotoxicity (MTS assay, flow cytometry analysis), values between groups were compared using two-way ANOVA (followed by Dunnett’s multiple analysis). The results were considered statistically significant when *p* value <0.05, <0.01, <0.001 and <0.0001.

### 2.9. Techniques

The morphology of SPIONs was investigated with Transmission Electron Microscopy (TEM), Zeiss Libra 120 Plus, Stuttgart, Germany, operating at 120 kV. Dynamic Light scattering (DLS) was used complementarily to TEM for analyzing the hydrodynamic size of SPIONs and hybrids. Experiments were carried out with Malvern Instruments Zetasizer Nano ZS, Malvern, UK. The material was also investigated by X-ray photoelectron spectroscopy (XPS) recorded on Kratos Axis Supra spectrometer (Swansea, UK), equipped with a monochromatic Al Ka radiation source (1486.7 eV). The instrument work function was calibrated to give a BE of 84.0 with 0.1 eV step for the 4f_7/2_ line of metallic gold, and the spectrometer dispersion was adjusted to give a BE of 932.62 eV for the Cu 2p_3/2_ line of metallic copper.

The modification of the SPIONs surface was characterized by FTIR spectroscopy with Nicolet 8700 Spectrometer Fisher Scientific. Thermogravimetric analysis (TGA) was performed with TGA Q50 (TA Instruments), New Castle, PA, USA, under a nitrogen atmosphere. The Magnetic hyperthermia (MH) measurements were performed with nanoScale Biomagnetics D5 Series equipment with CAL1 CoilSet. The Specific Absorption Rate (SAR) values were estimated using MaNIaC Controller software (nB nanoScale Biomagnetics, Zaragoza, Spain). Langmuir monolayers were prepared with a KSV-Nima KN2003 trough system with the KSV NIMA LB Software (Biolin Scientific, Manchester, UK).

## 3. Results and Discussion

### 3.1. Morphology Studies

**Figure 1:**
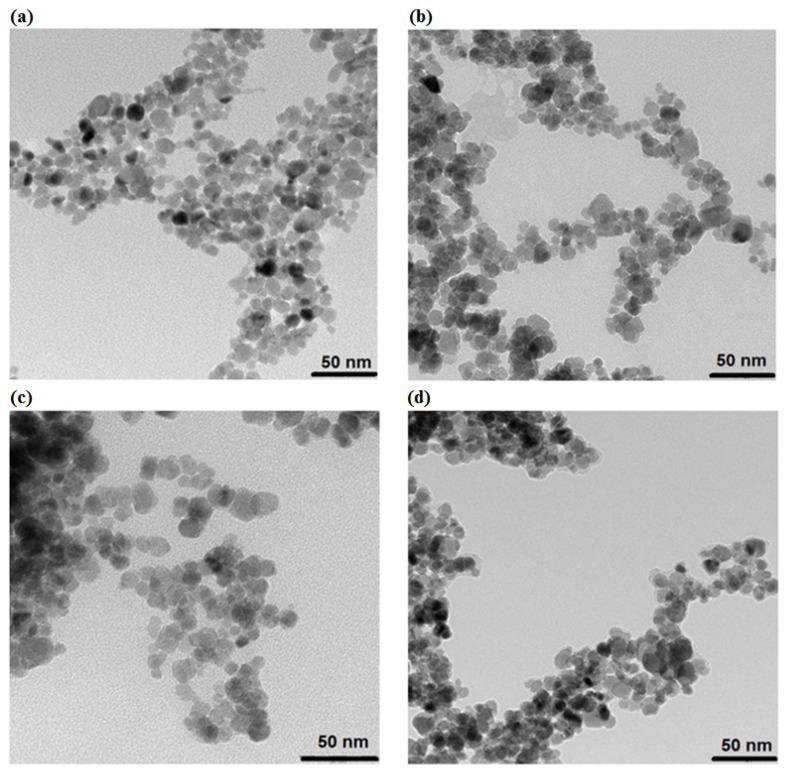
TEM images of particle size distributions (**a**) bare superparamagnetic iron oxide nanoparticles (SPIONs), (**b**) SPION@CA, (**c**) SPION@CA_Dox, and (**d**) SPION@CA_Epi (scale bar = 50 nm).

The studies of morphology were performed within the Transmission Electron Microscopy. The hybrid material (prepared with a coprecipitation technique, consisting of the spherical nanoparticles that have a size ranging from 10 to 15 nm) has a uniform surface and apparent aggregation. The aggregation that is presented on the TEM images is caused by the drying process onto a Formvar film covering the mesh for TEM analysis. As can be seen in Figure 1a, the SPIONs are having quite regular morphology and dispersity similar for the following samples. Figure 1b corresponds to the morphology studies of the SPIONs coated with citric acid (SPION@CA). The organic shell is too small to be clearly recognized. As can be seen, the morphology is nearby the same as for the sole SPIONs. Then the difference can be distinguished for SPIONs coated with citrates and loaded with an anticancer drug. Figure 1c shows the SPION@CA_Dox, while Figure 1d relates to SPION@CA_Epi. The superparamagnetic core can be clearly seen. The organic layer with loaded drugs for both TEM images Figure 1c,d are visible. Both doxorubicin and epirubicin have different densities than the magnetic core, so they as seen as the lighter layer coating core. The thickness of the organic shell is below 2 nm.

Additionally, to study the size of the prepared hybrid material, the Dynamic Light Scattering technique was used. The measurements for bare SPIONs were difficult to perform, due to the quick agglomeration. The results measured to coated SPIONs showed that an average hydrodynamic diameter for SPION@CA is about 27 ± 3 nm, while the values rise for anthracycline coating, see Figure S5. Hydrodynamic diameter for SPION@CA_Epi is about 69 ± 3 nm (Figure S6), while for SPION@CA_Dox is about 88 ± 6 nm (Figure S7). Along with DLS, the Zeta potential was measured for estimation of the surface potential, confirming the presence of functional groups on the surface of SPIONs.

Moreover, the Zeta potential was measured for estimation of the surface potential after SPIONs functionalization. For bare SPIONs, the Zeta potential was about –0.7 ± 0.03 mV. Negative values of the surface potential come from hydroxyl groups onto the SPIONs’ surface. The value is more negative for SPION@CA about –41.4 ± 3.1 mV, due to the carboxyl groups after SPIONs’ stabilization with citric acid. Values of Zeta potential for Epi and Dox hybrids are similar: For SPION@CA_Epi is about –25.2 ± 3.7 mV, while for SPION@CA_Dox, it is about: –26.5 ± 2.6 mV. Both, Dox and Epi were linked within the amide bond to citrates, so the values of Zeta potential for SPION@CA_Dox/Epi are less negative than SPION@CA.

### 3.2. XPS Analysis

**Figure 2:**
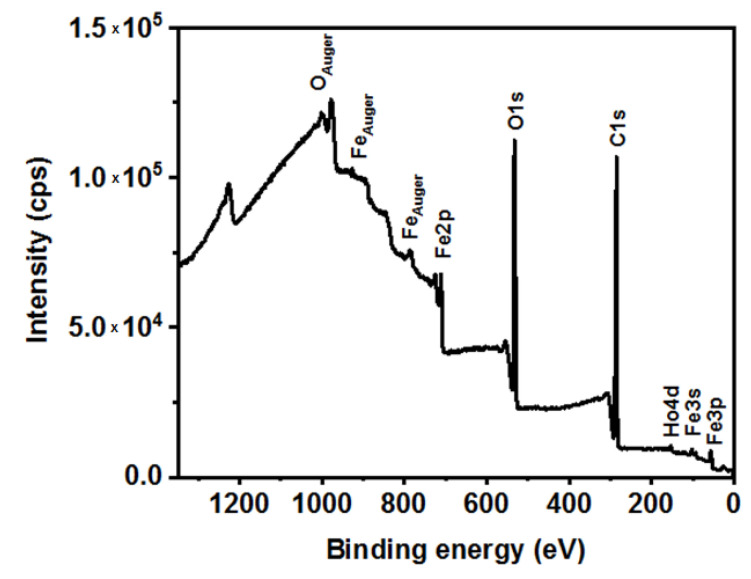
XPS overall spectrum of SPIONs doped with 1% holmium.

To investigate the chemical composition of as-synthesized SPIONs doped with 1% holmium the X-ray photoelectron spectroscopy was applied. The binding energy of Fe 2p and Ho 4d regions on XPS spectra was recorded. The overall spectrum of the naked SPIONs is presented in Figure 2.

**Figure 3:**
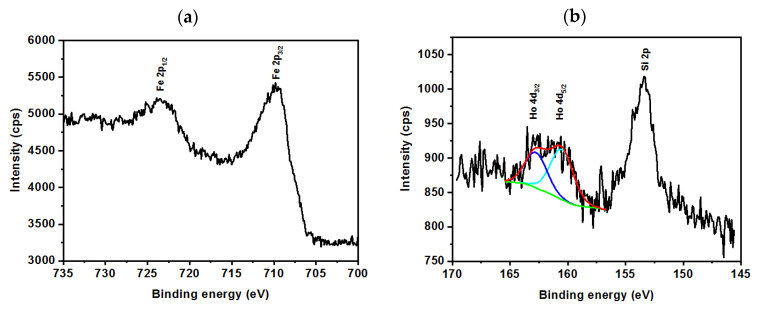
XPS spectrum for shows the region for (**a**) Fe 2p binding energies and (**b**) Ho 4d binding energies.

Following spectra presented in Figure 3a, shows the region characteristic to Fe 2p binding energies, where two peaks related to the Fe 2p_1/2_ about 723.6 eV and Fe 2p_3/2_ about 710.2 eV are clearly seen. These peaks are ascribed to Fe^3+^ ions occupying octahedral sites. The iron oxide results are in good agreement with the literature [55,56]. Figure 3b shows the spectrum at the range that is characteristic of the Ho 4d. As can be seen, two peaks for binding energies about 160.5 eV and 162.7 eV can be deconvoluted into peaks for Ho 4d_3/2_ and Ho 4d_5/2_. It can be ascribed to Ho^3+^ ions that are incorporated into the structure of iron oxide nanoparticles [57]. The following band of about 153.3 eV binding energy comes from the Si 2p present in the carbon-based tape containing a small number of additives like Si-based compounds, such as siloxane. The high noise in comparison to the signal for holmium is caused by the low Ho content in the samples. In this work, only about 1% molar was incorporated into the iron oxide lattice.

### 3.3. FT-IR Studies

**Figure 4:**
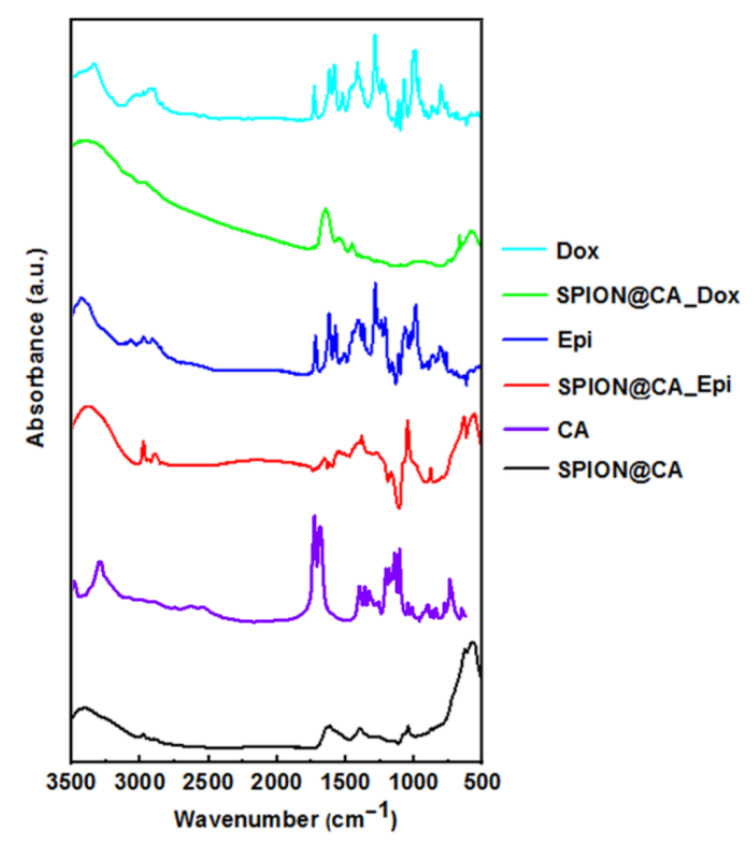
FT-IR spectra for Dox, Epi, SPION@CA, SPION@CA_Dox, and SPION@CA@Epi.

To investigate the conjugation of the SPIONs with CA, as well as drugs, the hybrid was studied with the use of Fourier Transformed Infrared Spectroscopy. The characteristic absorption spectra for Dox, SPION@CA_Dox, Epi, SPION@CA_Epi, and SPION@CA are presented in Figure 4. The peaks below 650 cm^−1^ are characteristic for Fe-O vibrations in the SPIONs’ lattice [58]. The following broad bands that are visible below 1000 cm^−1^ and 1395 cm^−1^ are characteristic for deprotonated carboxylic groups (COO^−^) for SPIONs stabilized with CA. It is in good agreement with the literature [59]. These bands are more intensive for CA modified SPIONs and the hybrid conjugates with Dox and Epi. Literature also indicates that the broad band of about 1600 cm^−1^ comes from the COO-Fe formation in the SPIONs [60]. Pure CA reveals characteristic bands of about 1711–1740 cm^−1^ that correspond to the stretching of the –COOH group. When the CA works as a linker, these bands are not observed.

Then, the bands of 1050 cm^−1^ and 1245 cm^−1^ characterize the oscillations of hydroxyl groups OH^−^ [61]. The bands of 1405 and 1550 cm^−1^ can be ascribed to the C-O vibrations at COO^−^ groups that are present in the hybrids loaded with drugs, while their intensity is much lower for SPION@CA_Dox and SPION@CA_Epi, which suggests linking of the carboxylic group from CA with amine bond in drugs.

According to the spectra of pure Dox and Epi, much more peaks are revealed. The band of 1380 cm^−1^ is assigned to the bending vibration of the alkyl group (CH_2_), while the presence of two peaks at 2840 cm^−1^ and 2910 cm^−1^ can be ascribed to the stretching vibration of the CH_2_. The bands that appear at 1276 cm^−1^ and 1210 cm^−1^ are derived from the vibration of enol (C–O). The band of 1632 cm^−1^ is characteristic for simultaneous stretching of C=C and C=O [62]; however, it can also be ascribed to the bending of the N–H groups [63].

The broad band of 3400 cm^−1^ that is present in all samples can be assigned to the stretching vibrations of the hydroxyl group, due to the humidity during the experiment, where the water vapors from the sample.

Within the analysis of the peak intensity in the range characteristic of the carboxyl group vibrations, as well as the range where the amine group vibrations, it is clearly seen that the spectra for SPION@CA and SPIONs loaded with drugs through the citric acid differ. The characteristic amide bond forms, which confirms the effective loading of the drug in the hybrid. Results are within good agreement with the literature [64].

### 3.4. Drug Content in Hybrid

**Figure 5:**
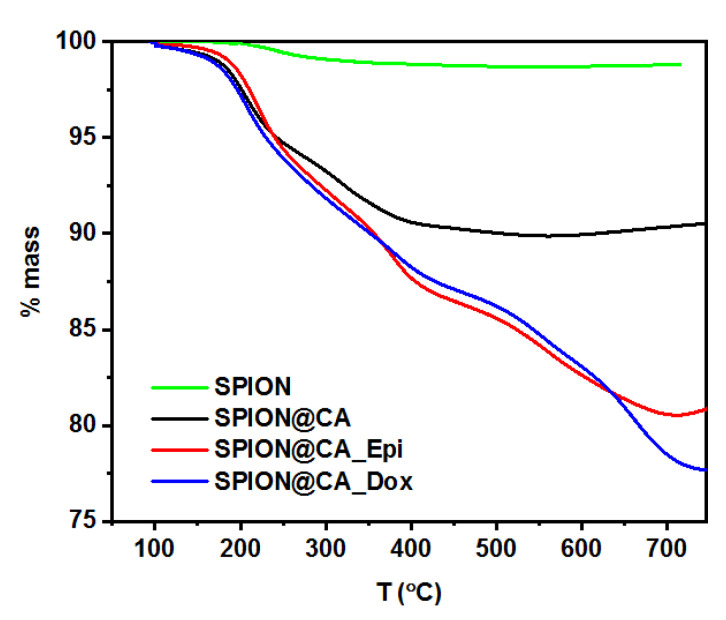
Thermograms of the SPION, SPION@CA, SPION@CA_Dox, and SPION@CA_Epi.

The amount of drug attached to the nanoparticles was determined by thermogravimetric measurement. After each stage of synthesis, the products (nanoparticles or conjugate) were tested using the TGA to compare results and estimate the average amount of the organic coating. This enabled the recording of TGA curves successively for unstabilized nanoparticles, citric acid-modified nanoparticles, and for conjugates. The TGA curves presented in Figure 5 illustrate the weight loss associated with the degradation of the organic substance attached to the nanoparticles. The thermogram performed for unmodified nanoparticles shows a slight weight loss of about 2%, which confirms the lack of an organic shell around the nanoparticles.

In the case of citric acid-modified SPIONs, it can be seen that the organic shell constitutes about 10% ± 1.0% by the weight of the entire carrier. Such an amount of citric acid is sufficient to stabilize SPIONs. On the basis of these data and the analysis of the TGA result of the synthesized conjugate, the amount of the drug attached to the nanoparticle can be determined.

It turns out that the content of the drug is 11 ± 1.0% and 9 ± 0.9%, for doxorubicin and epirubicin, respectively, which concludes that 110 ± 11 mg of doxorubicin and 90 ± 9 mg of epirubicin correspond to 1 g of SPIONs.

### 3.5. Investigation of Interactions of Hybrid with Biological Membranes

The Langmuir technique was used for the formation of analogs of the biological membrane under laboratory conditions. The conditions similar to the physiological environment were maintained to investigate the hybrid interactions with them [65].

#### 3.5.1. SPION@CA

The first stage of the research was to investigate the effect of nanoparticles on the monolayer formation process [66].

The negatively charged heads of cardiolipin interact with the negatively charged stabilizing shell of the SPION@CA, thereby causing mutual repulsion between the molecules. On the other hand, positively charged polar heads of DOPE attract nanoparticles and facilitate the penetration of the carrier into the monolayer. This kind of interaction is responsible for changing the angle of the molecules in the film, thus determining the value of surface area per phospholipid molecule in the monolayer. For the DOPC layer, the mentioned effect is insignificant. The addition of DOPE onto subphase increases the surface area occupied by one molecule in the layer that is associated with the concentration of nanoparticles. The presence of cardiolipin causes an increase in surface area per molecule in the membrane for concentrations ranging from 1–5 mg/L, while a decrease is observed for higher concentrations of nanoparticles. In both cases, these trends are more noticeable for a buffer with a pH of 5.8.

Regardless of the type of monolayer, the addition of a higher concentration of nanoparticles to the subphase does not change the way that carrier interacts with the lipids. Concentrations of nanoparticles equal 13 mg/L and 15 mg/L for experiments performed as preliminary experiments do not change the surface area per molecule in the monolayer and do not change the tilt of the particles in the membrane in relation to the carrier concentration of 10 mg/L.

In the next stage, the effect of drugs (one of the conjugate components) was investigated. These experiments are important for determining the influence of the entire conjugate and its components on the biomimetic membranes. Three different concentrations of drug solution were prepared: 1 × 10^−6^ M, 5 × 10^−6^ M, and 1 × 10^−5^ M correspond to the pharmacological concentrations used in vivo [67].

#### 3.5.2. Doxorubicin

For the obtained monolayers, the most pronounced increase in the surface area per molecule can be observed in the case of cardiolipin. It should be noted that for this lipid, even the lowest concentration causes changes in the surface area by 93% for a buffer with pH 5.8 and by 70% for a buffer with pH 7. It is worth mentioning that for both lipids: DOPC and DOPE, differences in the values of surface area between the lowest drug concentration and a pure buffer are 6% (DOPE) and 4.5% (DOPC) for a buffer at pH 5.8, and 16% (DOPE) and 2.2% (DOPC) for a buffer at pH 7. Cardiolipin is composed of two chains with a negative charge each, so it can attract two molecules of the drug, so the effect of the interaction is greater than for other lipids. The negatively charged polar heads interact with the drug to help it penetrate into the monolayer structure. This effect is more evident in a solution with a pH of 5.8, due to the positively charged amino groups in the drug structure [68].

#### 3.5.3. Epirubicin

For each layer formed on the subphase with the addition of epirubicin, the changes in surface area per molecule are observed. As mentioned earlier, the charged phospholipid heads interact with the drug to help it penetrate into the hydrophilic part of the membrane. Alike the case with doxorubicin, the most marked increase in surface area per molecule for cardiolipin was recorded. Mentioned changes are about 70% for buffer 5.8 and 35% for buffer pH 7.

#### 3.5.4. SPION@CA_Dox and SPION@CA_Epi

**Figure 6:**
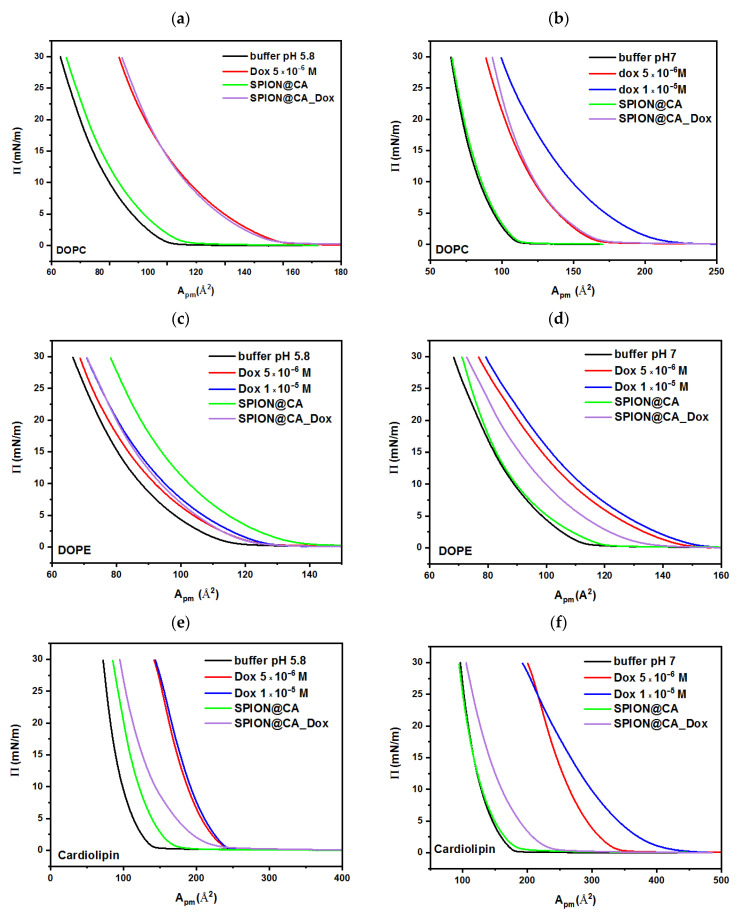
Langmuir isotherm for lipid (**a**,**b**) 1,2-dioleoyl-sn-glycero-3-phosphocholine (DOPC), (**c**,**d**) 1,2-dioleoyl-sn-glycero-3-phosphoethanolamine (DOPE), and (**e**,**f**) cardiolipin on phosphate buffer as a subphase with the addition of doxorubicin at two different concentrations, SPION@CA, and SPION@CA_Dox. The left column shows the results obtained at pH 5.8, while the right column presents the isotherms recorded at pH 7.

The last stage of the experiments was to study the influence of conjugates on the structure of biomimetic monolayers. The amount of carrier added to the subphase had to keep the drug concentration on the level 7.5 × 10^−6^ M. After the addition of the doxorubicin-conjugate to the subphase, the greatest changes are noticeable for the monolayer of cardiolipin. Based on the analysis of the π-A isotherm for cardiolipin, it can be assumed that the use of the conjugate reduces the interaction of the drug with the lipid membrane, limiting the penetration of the cytostatic. The shifts of the surface area per monolayer molecule relative to the area of the highest drug concentration are 33% for the buffer pH 5.8 and 45% for the buffer pH 7. For the other two lipids, the effect is negligible as there is no change between the isotherm recorded for the drug with the highest concentration and the isotherm of the conjugate for the pH 5.8 buffer, or the effect is very slight, around 6–7% for the pH 7 buffer, as shown in Figure 6.

**Figure 7:**
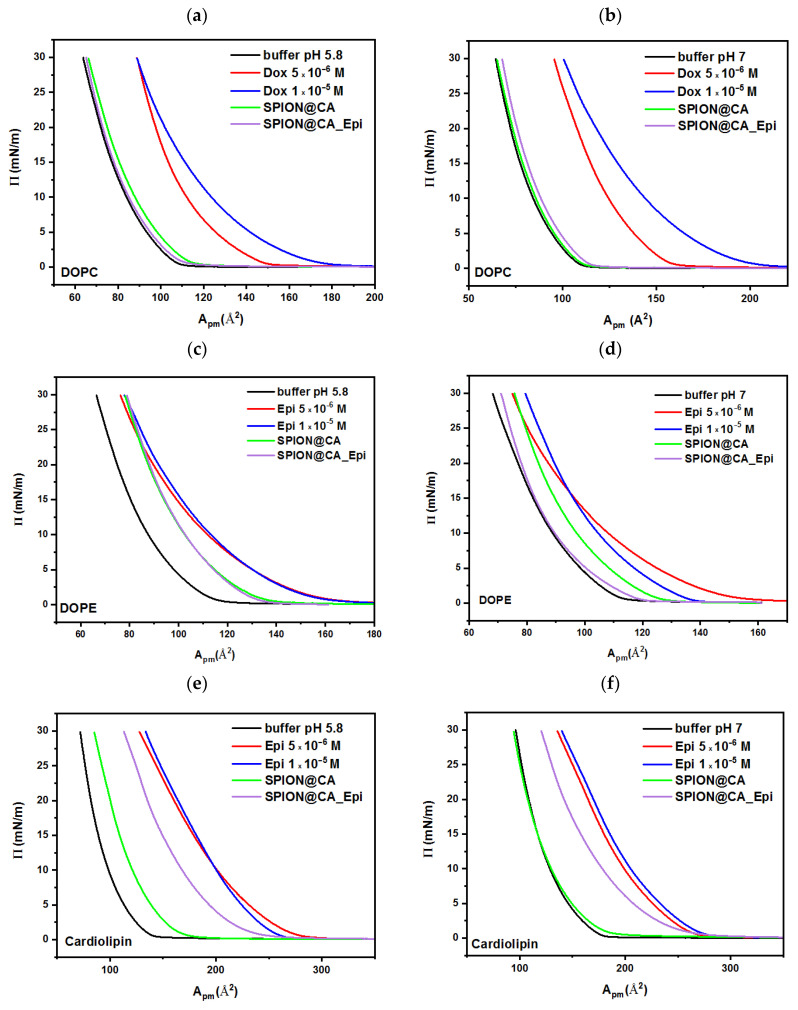
Langmuir isotherm for lipid (**a**,**b**) DOPC, (**c**,**d**) DOPE, and (**e**,**f**) cardiolipin on phosphate buffer as a subphase with the addition of doxorubicin at two different concentrations, SPION@CA, and SPION@CA_Epi. The left column shows the results obtained at pH 5.8, while the right column presents the isotherms recorded at pH 7.

When considering the π-A curves for SPION@CA_Epi, analog to the doxorubicin conjugate can be seen. For the DOPE curves, no significant changes in surface area per molecule in both buffers were observed. On the other hand, for both DOPC and cardiolipin, monolayer surface area decreased relative to the highest drug concentration at about 15% (cardiolipin) and about 30% (DOPC) in both environments in which the studies were performed. The use of the conjugate limited the penetration of antibiotics into a polar head group of lipids, which can be caused by the spatial location of the molecules and the lack of access to certain functional groups of the drug molecule that may interact with the membrane, see Figure 7 [60]. 

Conducted studies indicate that attaching the drug to a superparamagnetic carrier reduces its interaction with the biomimetic membrane, and thus, may affect the degree of anticancer drug accumulation in the membrane.

### 3.6. Magnetic Hyperthermia Studies

The heating efficiency of hybrids was studied by magnetic hyperthermia, where the magnetic energy of SPIONs is converted into heat under the application of the alternating magnetic field. The relaxation that generates the heat goes within the core within Néel and Brown modes [69]. Samples with a volume of about 0.5 mL and a density of 10 mg/mL were places in the copper coil, where they were thermostated. The experiments were performed with alternating magnetic field in the frequency range 386–633 kHz and with the amplitude up to 350 G. Measurements were performed until reaching 50 °C (323 K) in order to evaluate the time needed to achieve 42–46 °C, and followed an evaluation of the specific absorption rate (SAR).

Samples measured using MH had good colloidal stability, while after the measurements, the drug was released from the SPIONs’ surface. The measurements were performed in the phosphate buffer pH 5.8 (left column), mimicking tumor environment and human serum (right column). Acidic pH was chosen based on the literature that shows higher Dox release in the acidic pH than in neutral one [70,71].

**Figure 8:**
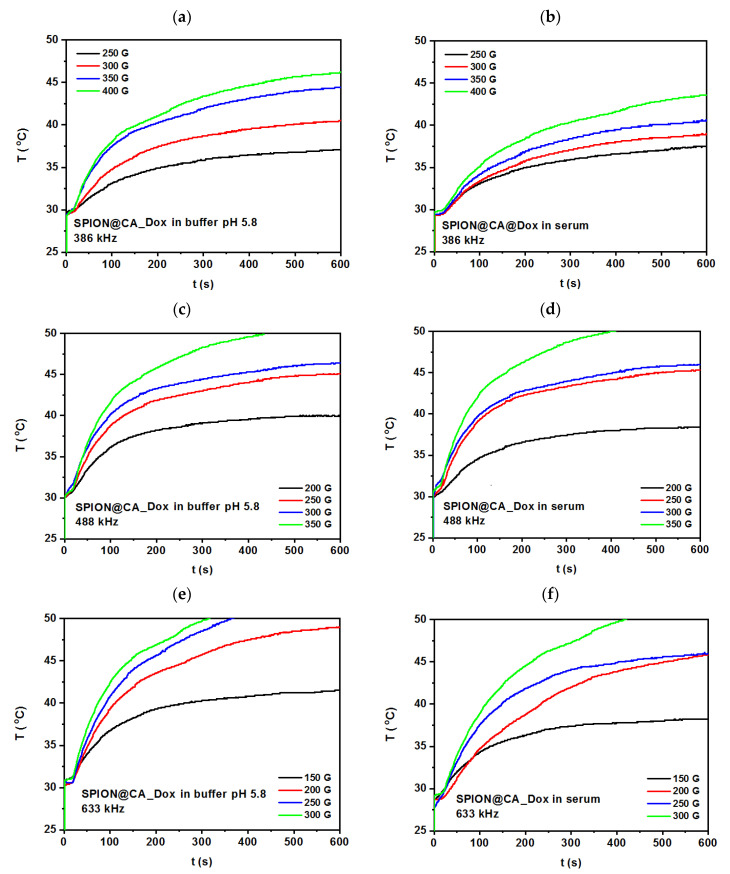
Temperature-time profile for hybrid SPION@CA_Dox measured at (**a**,**b**) at alternating magnetic field about 386 kHz, (**c**,**d**) 488 kHz, and (**e**,**f**) 633 kHz. The left column presents results for measurements performed in the phosphate buffer pH 5.8, while the right column corresponds to the experiments performed in the human serum.

**Figure 9:**
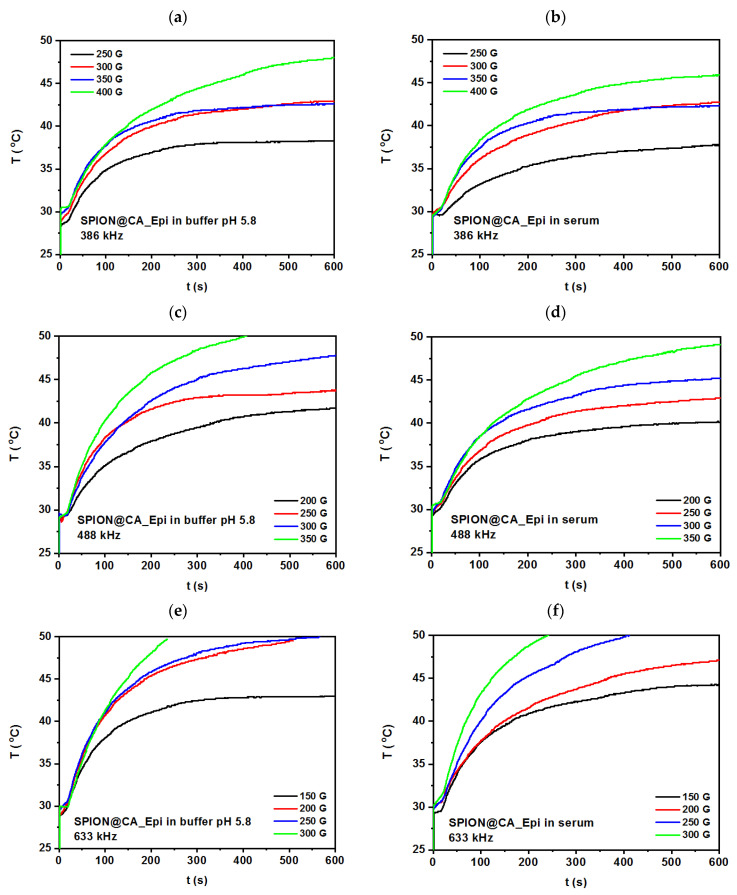
Temperature-time profile for hybrid SPION@CA_Epi measured at (**a**,**b**) at alternating magnetic field about 386 kHz, (**c**,**d**) 488 kHz, and (**e**,**f**) 633 kHz. The left column presents results for measurements performed in the phosphate buffer pH 5.8, while the right column corresponds to the experiments performed in the human serum.

As can be seen in Figure 8 and Figure 9, the alternating magnetic fields cause a spontaneous rise of the temperature in the colloidal suspension temperature, where after about 10 min, they tend to stabilize. Multiple studies show that the MH tissue treatment within that time is sufficient for therapy when achieved temperature reaches desired values [72]. These studies aimed to reach the temperature at about 42–46 °C in different media and compare how the serum that is more viscous than phosphate buffer influences the heating of the composite. For hybrid SPION@CA_Dox the frequency about 386 kHz, see Figure 8a,b shows that SPION@CA_Dox causes rise of the temperature rises to about 37 °C ± 1 °C, when the magnetic field amplitude is about 200 G, and the frequency is about 386 kHz. Almost the same values are reached in both media—while the 46 °C was achieved for the highest amplitude, about 400 G in buffer, and 43 °C for suspension measured in serum. In buffer media, 46 °C ± 2.4 °C was reached at amplitude about 400 G and 386 kHz. An increase in the amplitude causes the rise of the temperature, while for suspension measured in the serum, it is about 2 degrees lower than in buffer. The curve reaches plateau-like after a minimum of 5 min.

An increase of the frequency from 386 kHz to 488 kHz leads to the rise of temperature for about 5 degrees for 250 G, where the plateau starts to form at around 45 °C ± 2.3 °C for both 250 G and 300 G at both media—at buffer, and serum. As can be seen in Figure 8c,d, the curves recorded at both amplitudes are close to each other, while the amplitude of about 3500 G causes heating of colloidal suspension beyond 50 °C what excludes it from biomedical use. Following growth of the frequency from 488 kHz to 633 kHz requires the use of the amplitude below 300 G. Thus, the temperature for 300 G cause is much beyond the 50 °C, see Figure 8e,f showing HM curves for SPION@CA_Dox at both media and four amplitudes. Moreover, some fluctuations on the curve appear what corresponds to the possible agglomeration of colloidal suspension. Despite the narrow size distribution, when the amplitude is too high, nanoparticles may agglomerate, which results in nonhomogeneous heat generation of the colloidal suspension. The rise of temperature in serum is slightly smaller than in the phosphate buffer. So, the optimal parameters for further studies are in the range 250–300 G for 386–488 kHz, and about 250 G at 633 kHz. 

Following experiments were performed for SPION@CA_Epi at the same media—phosphate buffer and serum. The parameters applied to colloidal suspension during MH measurements were the same as for SPION@CA_Dox. As can be seen in Figure 9a,b, the plateau is reached at the same potential as the Dox-based hybrid, and it is about 37 °C ± 2 °C. However, the MH curve for 250 G and 350 G differs slightly, as presented in Figure 9a,b. These differences can be caused by slightly different drug content in the hybrid or nonhomogeneous linking of the drug with citric acid. The lower content of the drug is more visible on the curve recorded for 250 G, where they reached temperature is up to 2 °C higher than for SPION@CA_Dox hybrid. When the core has fewer chain-like structures, it more efficiently rotates in the AMF within the Brownian relaxation; what can be the possible reason for the higher temperature of colloidal suspension when the amplitude is higher. The lower temperatures for suspension in the serum are caused by higher viscosity in comparison to buffer, so hybrid has more interactions with viscous media what affects the heating efficiency.

Following measurements were performed for frequencies of about 488 kHz. As can be seen in Figure 9c,d, the temperatures reached after several minutes are higher in comparison to the SPION@CA_Epi. The same behavior is observed for the next curves recorded for 633 kHz. It is probably caused, as claimed above, by the lower content of the drug in the hybrid, so the mobility of the hybrid is faster. The heating is less effective in the serum, which correlates with higher viscosity of medium than for buffer. Based on these experiments, the optimal parameters for further studies on the animal model are about 250–300 G for 488 kHz and below 300 G for 633 kHz like for the hybrid SPION@CA_Dox.

Additionally, the specific absorption rate (SAR) was estimated for the linear range of the temperature in function of time (Figures S1−S4 and Tables S1 and S2). The average values for parameters (optimized using MH measurements, and performed in PBS and serum media) are presented in the Table 1 below.

The results clearly displace the difference between the dynamics of hybrid dispersed in less viscous PBS ad more viscous serum media. Additionally, as can be seen, the SAR values are only slightly higher for epirubicin. The SAR values are in good agreement with the results presented for iron oxide-based nanocomposites [73].

### 3.7. Cytotoxicity Results

**Figure 10:**
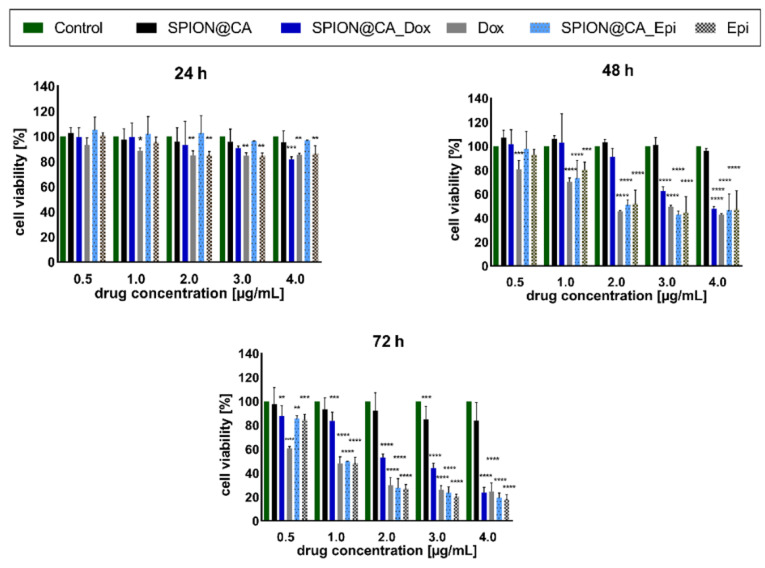
Cell viability of SKOV-3 cells treated with SPION@CA, SPION@CA_Dox, Dox, SPION@CA_Epi, and Epi after 24 h, 48 h, and 72 h. Nontreated cells were used as a control. Data are expressed as ± SD (*n* = 3). Statistical significance was considered if *p <* 0.05 (*), *p <* 0.01 (**), *p <* 0.001 (***) and *p <* 0.0001 (****).

To evaluate the cytotoxicity of NPs conjugated with Dox/Epi in vitro cell studies using MTS reagent were performed, see Figure 10. Obtained results showed that SPIONs coated with citric acid show no to little cytotoxicity, since more than 80% of cells survived after 72 h. It can also be noted that SPION@CA_Dox, Dox, SPION@CA_Epi, and Epi cytotoxicity increased in a time and concentration-dependent manner. SPION@CA_Dox significantly reduced viability of SKOV-3 cells up to the concentration of 3 µg/mL (0.3 µg/mL Dox) after 48 and 72 h of incubation time. Furthermore, SPION@CA_Dox at the concentration of 4 µg/mL causes similar cytotoxicity as DOX, after 72 h. The IC_50_ values for SPION@CA, SPION@CA_Dox, Dox, SPION@CA_Epi_and Epi determined after 72 h were 4.7 ± 0.4, 2.4 ± 0.5, 0.9 ± 0.3, 1.2 ± 0.2, and 1.1 ± 0.4 µg/mL, respectively. The results indicate that free DOX is more toxic in all presented concentrations than this chemotherapeutic attached to SPIONs. On the contrary, this effect was not observed for SPION@CA_Epi/Epi, which showed both similar cytotoxicities regardless of concentration and incubation time.

### 3.8. Apoptosis

**Figure 11:**
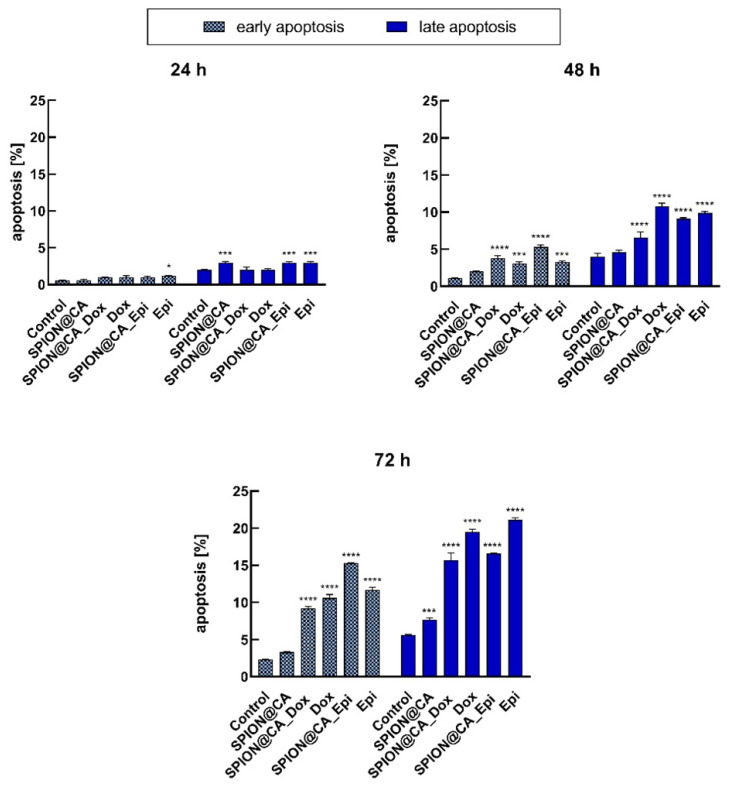
Percentage of apoptotic cells assessed by cytometry on SKOV-3 cells treated with SPION@CA, SPION@CA_Dox, Dox, SPION@CA_Epi, and Epi after 24 h, 48 h, and 72 h. Nontreated cells were used as a control. Data are expressed as ± SD (*n* = 3). Statistical significance was considered if *p <* 0.05 (*), *p <* 0.001 (***) and *p <* 0.0001 (****).

To determine the cytotoxicity of the compounds, apoptosis of SKOV-3 cells was analyzed by flow cytometry using Annexin V FITC and PI fluorescence staining assay. Based on MTS results, the highest concentration of SPION@CA (4 µg/mL) and Dox/Epi (0.4 µg/mL) was chosen. Overall, tested compounds induced mostly late apoptosis to a greater degree than early apoptosis, and this trend was maintained at each time point, as shown in Figure 11. After 24 h only 0.6–3.7% of apoptotic cells were detected, whereas longer incubation time caused higher apoptosis (3.3–10.8% after 48 h and 2.3–21.1% after 72 h, respectively). The percentage of apoptotic cells of SPION@CA was comparable to control cells. The apoptosis induced by DOX and Epi chemotherapeutics was similar. The treatment of SKOV-3 cells with DOX was more effective than with SPION@Dox leading to the increase in late apoptotic cells (15.7% via 19.5% after 72 h). In the case of SPION@Epi/Epi, a comparable percentage of apoptosis was observed, however, contrary to SPION@Epi; Epi treatment led to more late apoptotic cells. Presented results are comparable to our viability studies, where colorimetric MTS assay was used.

## 4. Discussion

We report a nanoformulation and investigation of hybrid material based on holmium doped SPIONs loaded with doxorubicin or epirubicin. SPIONs were obtained with the coprecipitation technique, where the holmium 1% by molar ratio was incorporated into the superparamagnetic core.

Then, the SPIONs were modified with citric acid for core protection against agglomeration and further linking with anticancer drugs. Citric acid coating SPIONs constitute about 10% of the mass. Such an amount is sufficient for the effective stabilization of bare nanoparticles. TEM studies confirm that obtained SPIONs reveal spherical shape and size ranging from 10 to 15 nm, while the citric acid coating is not visible well onto the surface. However, the FT-IR spectra confirm successful coating with citric acid along with Zeta potential studies. The surface potential for bare SPIONs is below –1.0 mV, while for SPION@CA is about –41.4 ± 3.1 mV suggesting the presence of negatively charged functional groups onto the SPIONs surface. In the case of bare SPIONs, only some hydroxyl groups are present, while for SPION@CA the negative charge comes from carboxylic groups COO^−^.

Then, two drugs were linked to SPION@CA within the amide bond by use of EDC and NHS agents. Both drugs were successfully linked to citric acid forming SPION@CA_Dox and SPION@CA_Epi hybrids, where Epi constituted about 9% by mass and doxorubicin 11% by mass of hybrid, respectively. As cytotoxicity studies show, these amounts were sufficient for effective toxicity against cancer cells. The modification of SPION@CA with drugs decreases the Zeta potential values for about −25.2 mV ± 3.7 for SPION@CA_Epi and about −26.5 mV for SPION@CA_Dox. That confirms successful conjugation of SPION@CA with drugs.

Additionally, hybrids were placed on the analogs of biological membranes to check their interactions with lipids. Langmuir isotherms prove that anthracyclines have a high affinity for some types of lipids, but the way the drug is bound to the carrier may significantly reduce this effect. The greatest accumulation of both drugs in the membrane occurs for the lipid—cardiolipin. That lipid is present mainly in the membranes of mitochondrial or cardiac muscle cells, which may explain the high level of anthracycline accumulation in these cells in the body. Conjugation of the drug with a magnetic carrier can not only be used in targeted therapy, but it can also reduce the amount of anthracycline that enters and accumulates in cells, such as the heart muscle.

The resultant hybrid can be easily synthesized, and its properties can be modified within experimental conditions controlling their amount desire for single preparation, size, and shape of the magnetic core, as well as the anticancer drug loading into the hybrid. The drug release can be induced by AMF, and hybrid offers its application in magnetic hyperthermia. The drug released from SPIONs’ surface crosses biological membranes. The heating of hybrid depends on the viscosity of the media in which it is suspended. In the serum the heating is about 2 °C lower than in the PBS. To achieve desired 42–46 °C, the application of the frequency of about 488 kHz and amplitude of the magnetic field in the range 250–300 G is required. For a higher frequency, like 633 kHz, the 250 G amplitude is sufficient.

The cytotoxicity is an important factor in evaluating the potential of designed drugs in cancer therapy. The results obtained from biological studies indicate that SPION@CA_Dox and SPION@CA_Epi exhibit cytotoxicity towards cancer cells. The toxicity depends on the used concentrations of SPION@CA and Dox/Epi and also incubation time. No higher effect of chemotherapeutics conjugated to SPIONs in comparison to free cytostatics was found, as it is usually seen in literature [74]. Additionally, even lower cytotoxicity of Dox loaded SPIONs comparable to free Dox was also observed (as well as in our studies), due to nonimmediate exposure to chemotherapeutic. Furthermore, examination of annexin V and propidium iodide revealed a smaller proportion of cells undergoing early apoptosis and clear detection of late apoptotic/necrotic cells. The results of flow cytometry are in good agreement with cytotoxicity studies performed by MTS assay and confirm the effectiveness of synthesized compounds.

## 5. Conclusions

In conclusion, the hybrids, based on holmium-doped SPIONs were synthesized and linked with citric acid and the anticancer drugs like doxorubicin and epirubicin. SPIONs core has a spherical shape and size of about 15 nm. Magnetic hyperthermia investigation reveals that it is facile to moderate the heating within experimental conditions with maintaining desired SAR values. Colloidal suspension can be easily heated up to desired 42–46 °C in about 10 min when the frequency of AMF is about 488 kHz and amplitude 250–300 G or lower amplitude for a higher frequency. The hybrids studies with the Langmuir trough technique indicate that hybrids interact with biological membranes analogs mainly through electrostatic interactions.

Cytotoxicity studies show that our synthesized drugs based on Dox and its less cardiotoxic analog, Epi, were successfully effective towards cancer cells. Nevertheless, further in vitro biological studies performed on three-dimensional cell cultures—spheroids (3D), as well as in vivo experiments are required to evaluate their potential in cancer treatment. Additionally, the conjugation of NPs to targeting biomolecules would allow for more selective and efficient drugs for targeted therapy.

To summarize, this work presents the features of the hybrids SPION@CA_Dox and SPION@CA_Epi as promising materials for anticancer therapy.

## Figures and Tables

**Table 1:** Specific absorption rate (SAR) values for SPION@CA@Dox and SPION@CA_Epi hybrids.

SPION@CA_Dox			
PBS pH 5.8	250 G	488 kHz	258.9 W·g^−1^
Serum	250 G	488 kHz	224.7 W·g^−1^
PBS pH 5.8	300 G	488 kHz	322.1 W·g^−1^
Serum	300 G	488 kHz	306.8 W·g^−1^
**SPION@CA_Epi**			
PBS pH 5.8	250 G	488 kHz	283.5 W·g^−1^
Serum	250 G	488 kHz	256.4 W·g^−1^
PBS pH 5.8	300 G	488 kHz	369.7 W·g^−1^
Serum	300 G	488 kHz	337.3 W·g^−1^

## Data Availability

Data can be obtained by contacting authors.

## Supplementary Materials

The following are available online at https://www.mdpi.com/article/10.3390/pharmaceutics13040480/s1, Figure S1. SPION@CA_Epi conjugates measured in buffer solution pH 5.8 with frequency about 488 kHz. Figure S2. SPION@CA_Epi conjugates measured in serum with frequency about 488 kHz. Figure S3. SPION@CA_Dox conjugates measured in buffer solution pH 5.8 with frequency about 488 kHz. Figure S4. SPION@CA_Dox conjugates measured in serum with frequency about 488 kHz. Table S1. SPION@CA_Epi conjugate. Table S2. SPION@CA_Dox conjugate. Figure S5. Original DLS graph recorded for SPION@CA. Figure S6. Original DLS graph recorded for SPION@CA_Epi. Figure S7. Original DLS graph recorded for SPION@CA_Dox.
