# Supplementary material for: Hybrid System for Local Drug Delivery and Magnetic Hyperthermia Based on SPIONs Loaded with Doxorubicin and Epirubicin
Source: Pharmaceutics. 2021 Apr 1;13(4):480. doi: 10.3390/pharmaceutics13040480 (PMC8066659; doi:10.3390/pharmaceutics13040480)
Supplement: Supplementary file 1 [file pharmaceutics-13-00480-s001.pdf]

pharmaceutics

MDPI

Supplementary Materials: Hybrid System for Local Drug Delivery and Magnetic Hyperthermia Based on SPIONs Loaded with Doxorubicin and Epirubicin

Dorota Nieciecka, Joanna Celej, Michał Żuk, Agnieszka Majkowska-Pilip, Kinga Żelechowska-Matysiak, Antoni Lis and Magdalena Osial

Figures present thermograms of samples for which SAR values were measured. Curves were fitted to the typical exponential function (Equation (S1)) described in the literature [1]:

$$\Delta T = a\,(1 - e^{-bt}) \qquad \text{(S1)}$$

Parameters **a** and **b** of matched functions are presented in tables 1 and 2. Parameter **a** has interpretation of equilibrium temperature for a time approaching infinity, while parameter **b** determines the rate of approaching temperature.

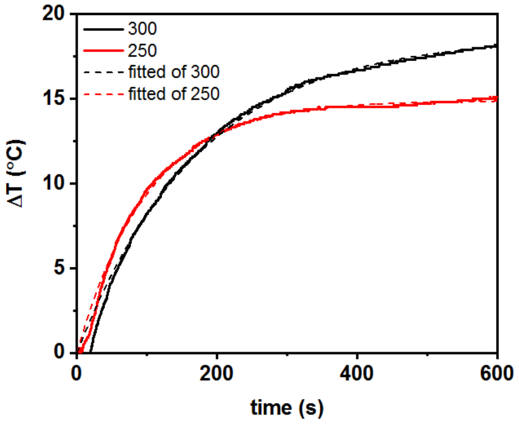

Figure S1. SPION@CA_Epi conjugates measured in buffer solution pH 5.8 with frequency about 488 kHz.

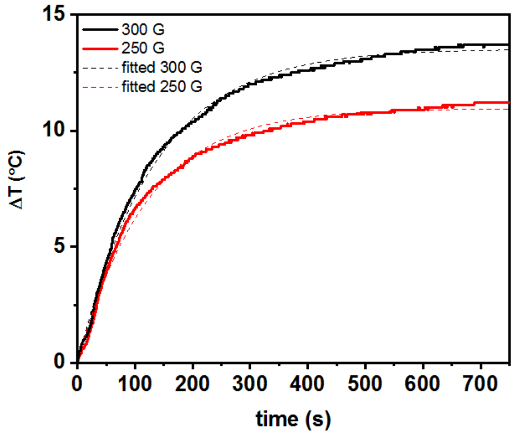

Figure S2. SPION@CA_Epi conjugates measured in serum with frequency about 488 kHz.

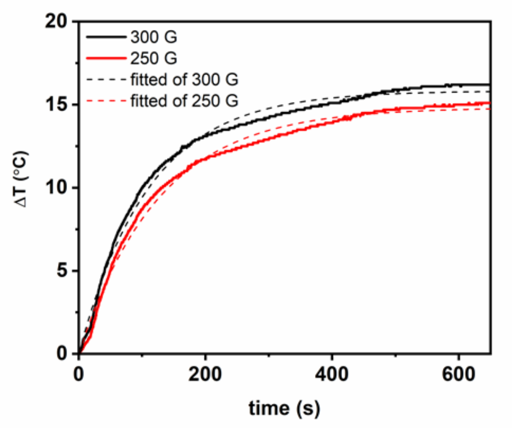

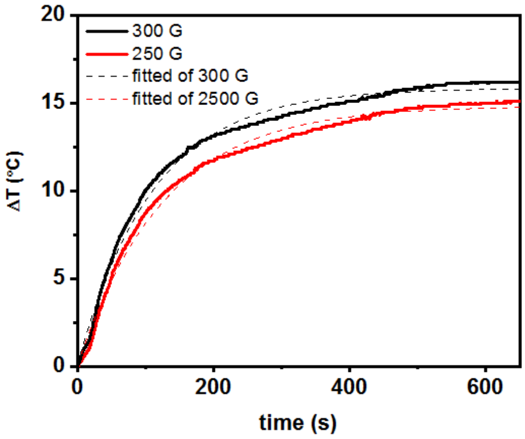

Figure S3. SPION@CA_Dox conjugates measured in buffer solution pH 5.8 with frequency about 488 kHz.

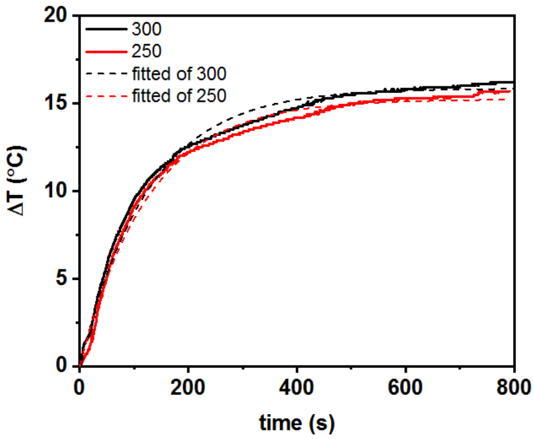

Figure S4. SPION@CA_Dox conjugates measured in serum with frequency about 488 kHz.

Table S1. SPION@CA_Epi conjugate.

Solution	Magnetic Field	Par. a	Par. b
buffer	250	14.89868	0.00994
buffer	300	18.64557	0.00577

serum	250	10.95956	0.00838
serum	300	13.52571	0.00752

Table S2. SPION@CA_Dox conjugate.

Solution	Magnetic Field	Par. a	Par. b
buffer	250	14.82617	0.00793
buffer	300	15.82997	0.00902
serum	250	15.23439	0.00802
serum	300	15.8557	0.00801

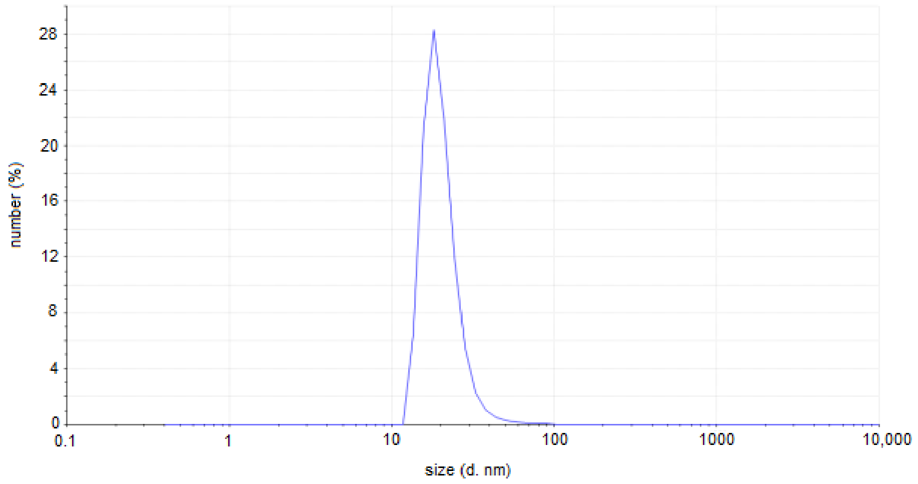

Figure S5. Original DLS graph recorded for SPION@CA.

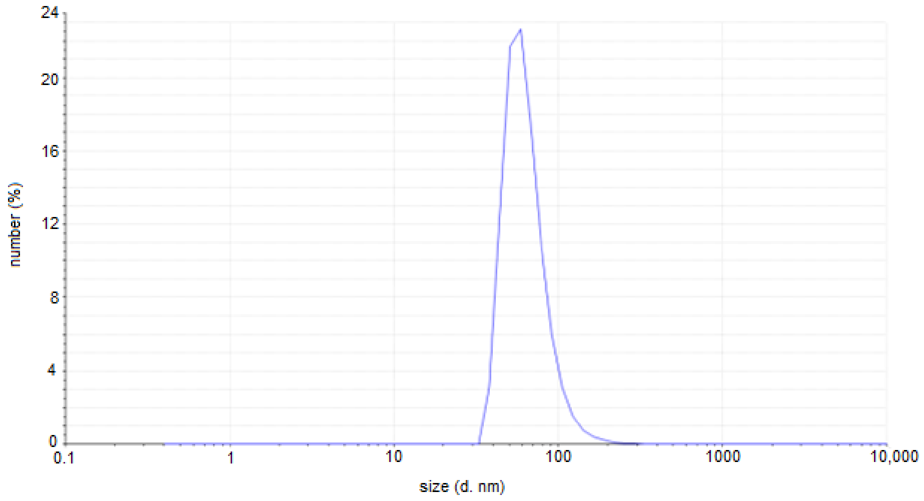

Figure S6. Original DLS graph recorded for SPION@CA_Epi.

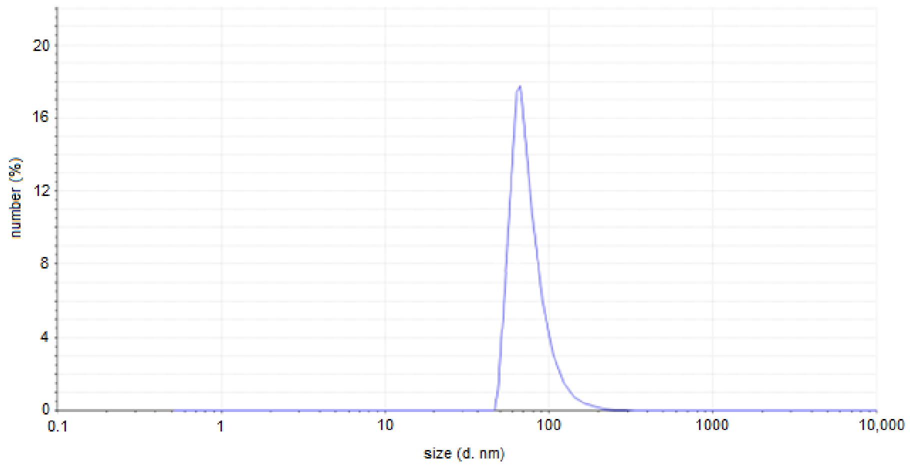

Figure S7. Original DLS graph recorded for SPION@CA_Dox.

References

1. Ebrahimisadr, S.; Aslibeiki, B.; Asadi, R. Magnetic Hyperthermia Properties of Iron Oxide Nanoparticles: The Effect of Concentration. *Phys. C Supercond. Its Appl.* **2018**, *549,* 119–121, doi:10.1016/j.physc.2018.02.014.
